# A Point-of-Care Immunosensor for Human Chorionic Gonadotropin in Clinical Urine Samples Using a Cuneated Polysilicon Nanogap Lab-on-Chip

**DOI:** 10.1371/journal.pone.0137891

**Published:** 2015-09-14

**Authors:** S. R. Balakrishnan, U. Hashim, Subash C. B. Gopinath, P. Poopalan, H. R. Ramayya, M. Iqbal Omar, R. Haarindraprasad, P. Veeradasan

**Affiliations:** 1 Biomedical Nano Diagnostics Research Group, Institute of Nano Electronic Engineering (INEE), Universiti Malaysia Perlis (UniMAP), Kangar, Perlis, Malaysia; 2 School of Microelectronic Engineering, University Malaysia Perlis (UniMAP), Kuala Perlis, Perlis, Malaysia; 3 Department of Obstetrics and Gynaecology, Hospital Tuanku Fauziah, Kangar, Perlis, Malaysia; 4 University Health Centre, Universiti Malaysia Perlis (UniMAP), Kuala Perlis, Perlis, Malaysia; Queen's University at Kingston, CANADA

## Abstract

Human chorionic gonadotropin (hCG), a glycoprotein hormone secreted from the placenta, is a key molecule that indicates pregnancy. Here, we have designed a cost-effective, label-free, *in situ* point-of-care (POC) immunosensor to estimate hCG using a cuneated 25 nm polysilicon nanogap electrode. A tiny chip with the dimensions of 20.5 × 12.5 mm was fabricated using conventional lithography and size expansion techniques. Furthermore, the sensing surface was functionalized by (3-aminopropyl)triethoxysilane and quantitatively measured the variations in hCG levels from clinically obtained human urine samples. The dielectric properties of the present sensor are shown with a capacitance above 40 nF for samples from pregnant women; it was lower with samples from non-pregnant women. Furthermore, it has been proven that our sensor has a wide linear range of detection, as a sensitivity of 835.88 μA mIU^-1^ ml^-2^ cm^-2^ was attained, and the detection limit was 0.28 mIU/ml (27.78 pg/ml). The dissociation constant K_d_ of the specific antigen binding to the anti-hCG was calculated as 2.23 ± 0.66 mIU, and the maximum number of binding sites per antigen was B_max_ = 22.54 ± 1.46 mIU. The sensing system shown here, with a narrow nanogap, is suitable for high-throughput POC diagnosis, and a single injection can obtain triplicate data or parallel analyses of different targets.

## Introduction

Human chorionic gonadotropin (hCG) is a hormone that is expressed by placental trophoblasts during pregnancy and comprises α and β-subunits (hCGα & hCGβ). hCG has been widely considered as a biomarker of pregnancy and can protect against complications during prenatal care. It is also an important and critical marker in many advanced malignancies and can assist in cancer detection. Further, complications such as ectopic pregnancy, threatened abortion and pregnancy trophoblastic diseases, such as acephalocystis racemosa and chorioepithelioma, could be monitored via the detection of hCG [1–3]. This monitoring can be performed by antibody-assisted biosensors, or ‘immunosensors’, which have been developed using different types of electrodes to recognize specific targets on the sensing surface of biochips [4,5]. There are other sensing systems or strategies that have also been reported with high performance in hCG measurements [6–17]. Furthermore, different methods for hCG detection have been proposed using lab-dependent systems, such as surface plasmon resonance [6,18], enzyme-linked immunosorbent assay (ELISA) [19,20], fluoroimmunoassay [21], radioimmunoassay [22], immunochromatographic assay [23,24], resonance scattering spectral [25] and other sensing systems, including electrochemical [7,12–15,17,26,27], amperometric [16,28,29], capacitive [30] and impedimetric [31,32] systems. However, developing a lab-independent electrochemical immunosensor is necessary to facilitate the replacement of current existing lab-dependent detection systems.

In most instances, lab-dependent assays require a large working space and long durations and involve the use of chemicals, which slows down the result acquisition processes. The development of independent point-of-care (POC) electrochemical immunosensors will overcome these limitations and promises a number of significant advantages, including simplicity, fast response, suitability for onsite testing, high sensitivity and time and cost savings. Consequently, the frequency of hospital visits, travel expenses and lost productivity on the job could be reduced. The success of ‘bedside analyzing systems’, such as glucose meters, has promoted the development of similar quantitative biosensing devices for pregnancy, which rely on myriad detection schemes. One of the promising alternatives to existing biosensors is capacitive based and can be used for POC applications. Capacitive biosensors are favored due to their ability to detect the analyte directly in a sample with little or no preparation [33]. The capacitive behavior of this type of sensor is based on parameters such as changes in dielectric properties, charge distribution, permittivity and conductivity that originate from the complex matrix formation on the surface of the transducing electrodes. Hence, changes in such parameters could be measured when biomolecule binding occurs on the electrode surface. In the quest for higher sensitivities and more accurate measurements, the sensing electrode shown in the present study could be used as a capacitive sensor.

To develop the sensor, we used standard semiconducting materials, with silicon as the substrate and polysilicon (PS) as the sensing electrode. Polysilicon was chosen for its ability to withstand high temperatures and its high compatibility with standard semiconductor processing steps. The advantages of this material also include high sensitivity, a fast response time and high accuracy, as previously reported [34,35]. In the quest to reduce the use of expensive equipment, a conventional lithography microfabrication technique was implemented; this technique can be used to fabricate simple and non-complex nanostructures such as nanogaps and nanowires [36,37]. In this study, we developed a narrow-sized nanogap-based sensor with a single injection capability to obtain triplicate data or parallel analyses against different targets. Furthermore, because it is possible to block unused channel(s) to limit the sample usage, this system is more cost effective and easier to use. All abbreviation and symbols used in this study are described in Table 1.

**Table 1:** List of Symbols and Abbreviation.

PSNG	Polysilicon Nanogap
PS	Polysilicon
hCG	Human Chorionic Gonadotropin
A	Electrode area
ν	Applied voltage
l_g_	Gap width
l_e_	Electrode width
l_SM_	Width of total surface modification
l_hcg_	hCG width
ρ_air_	Air resistivity
ρ_e_	Electrode resistivity
ρ_SM_	Resistivity of total surface modification
P_hCG_	hCG resistivity
ɛ_T_	Total permittivity
ɛ_e_	Permittivity of electrode
ɛ_SM_	Permittivity of total surface modification
ɛ_hCG_	Permittivity of hCG
Z_T_	Total impedance
d_e_	Dielectric width of electrode
d_SM_	Dielectric width of total surface modification
d_hcg_	Dielectric width of hCG

## Materials and Methods

### Materials and Reagents

Single crystal silicon wafers were used as substrates for the lab-on-chip (LOC) development throughout the experiment. The fabrication of polysilicon nanogap (PSNG) electrodes requires cleaning of the wafers using buffered oxide etchant (BOE; 6:1; J.T. Baker, Center Valley, Pennsylvania, USA) and standard cleaning 1 (RCA1) and standard cleaning 2 (RCA2) solutions. Positive photoresist (PR1-2000A), negative photoresist (NR7-6000PY) and a resist developer (RD6) for pattern transfer were purchased from Futurrex, Inc., Franklin, New Jersey, USA. Acetone, aluminum etch (80-15-3-2), hydrochloric acid (HCl; 37%), aqueous ammonia (NH_4_OH; 30%), hydrogen peroxide (H_2_O_2_; 30%), and sulfuric acid (H_2_SO_4_) were purchased from J.T. Baker, Center Valley, Pennsylvania, USA. The DI water used throughout the experiment was produced by an RO deionization system (13–16 MΩ-cm, SASTEC (M) Sdn. Bhd, Malaysia). Aluminum (Al), titanium (Ti) and gold (Au) metals were used to deposit thin Al, Ti and Au layers. Microfluidic fabrication was performed using the Sylgard 184 Silicone Elastomer Base and Sylgard 184 Silicone Elastomer Curing Agent (Dow Corning Corp., Midland, USA) with SU-8 negative photoresist and isopropanol (IPA) from J.T. Baker, Center Valley, Pennsylvania, USA. To achieve surface modification, iron (II) sulfate (FeSO_4_), sodium bicarbonate (NaHCO_3_) and chloroform (CHCl_3_) were purchased from HmBG Chemicals, Germany. (3-aminopropyl)triethoxysilane (APTES) 99% of 221.37 g/mol, bovine serum albumin (BSA) ≥98.0%, trifluoroacetic acid (TFA) 99% CF_3_CO_2_H, N-(3-dimethylaminopropyl)-N′-ethylcarbodiimide hydrochloride (EDC), di-tert-butyl dicarbonate (tBOC; ≥99%) C_10_H_18_O_5_, N-hydroxysuccinimide (NHS; 98%) C_4_H_5_NO_3_, ethylenediaminetetraacetic acid (EDTA; ≥99%) C_10_H_16_N_2_O_8_ and phosphate buffered saline (PBS; pH 7.4) were purchased from Sigma-Aldrich, St. Louis, Missouri, USA. Glucose (GLUC), carcinoembryonic antigen (CEA), uric acid (UA) and ascorbic acid (AA), also from Sigma-Aldrich, St. Louis, Missouri, USA, were used for the selectivity test. Monoclonal hCG beta antibody (hCGab; GTX42543; GeneTex, Irvine, California, USA) and hCG standards were tested with the hCG ELISA Kit; CSB-E05060h; CUSABIO Biotech, Hubei, China. All fresh 24-h urine samples for pregnant and non-pregnant women were obtained with permission from the UniMAP Health Centre. All samples were acquired with unrecorded verbal consent from patients who were informed about the research purposes, and this consent procedure was approved by the Medical Ethics Committee for the University Health Centre, Universiti Malaysia Perlis. The above-mentioned ethics committee specifically approved this study, which was conducted in accordance with the International Conference on Harmonisation–Good Clinical Practice (ICH–GCP) guidelines and the Declaration of Helsinki.

### Design and fabrication of PSNG lab-on-chip (LOC)

**Fig 1:**
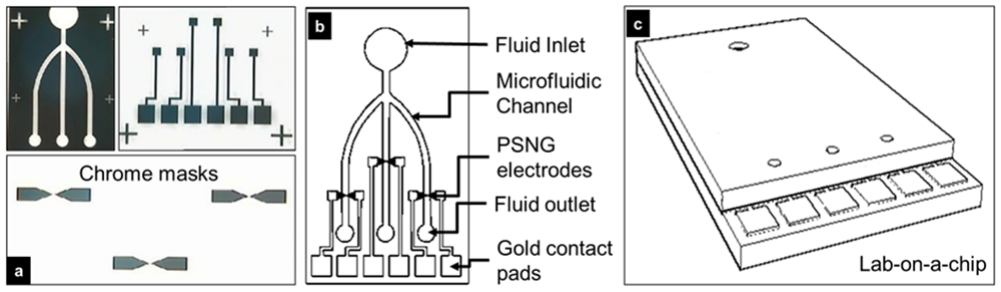
Design and specifications of the LOC immunosensor. (a) The chrome mask, as seen under microscopy, for the electrodes, contacts and microfluidics used for the sensor fabrication. (b) The specifications of the immunosensor consisting of microfluidic channels for fluid delivery and chemically modified PSNG electrodes underneath for hCG detection. (c) The designed LOC immunosensor with dimensions of 20.5 × 12.5 mm.

The patterns for the PSNG electrode, contact pads and microfluidics were designed in AutoCAD for pattern transfer via mask aligner (Fig 1a). The PSNG mask has a critical dimension of 1 μm at the electrode gap. The 20.5 mm × 12.5 mm LOC immunosensor is shown in Fig 1b. The LOC consists of three standalone PSNG electrodes as the base and is bonded with microfluidic channels that serve as the fluid delivery system to the specific electrodes (Fig 1c) [38–47].

**Fig 2:**
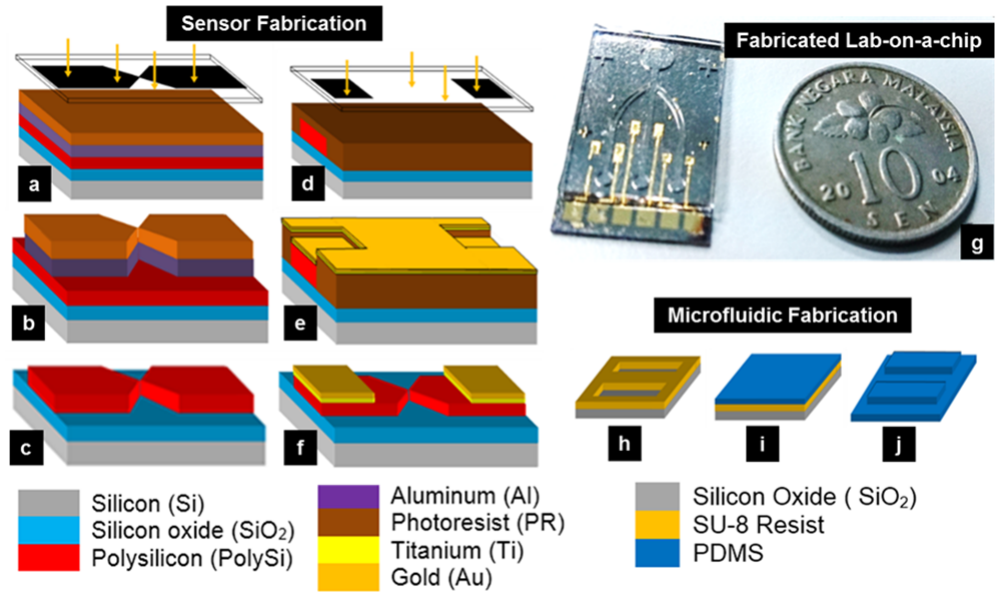
Fabrication of the PSNG LOC immunosensor using standard conventional lithography. (a-f) PSNG electrode fabrication using electrode and contact chrome masks. (g) Final fabricated LOC. Size comparison is shown with a coin. (h-j) Soft lithography using a microfluidic chrome mask for fluid sample delivery.

A conventional lithography technique was used to fabricate the PSNG immunosensor, as reported elsewhere [37,43]. Fig 2a–2f explains the simplified fabrication process steps used to develop the PSNG electrode. Oxide was grown by wet oxidation, and polysilicon (PS) was deposited by the low-pressure chemical vapor deposition (LPCVD) technique. The deposited PS wafer was then annealed for 12 h at 600°C to allow the disordered PS molecules to rearrange after deposition. Al was first deposited as a sacrificial layer and patterned using the MIDAS Exposure System MDA-400M for the first electrode mask before PS etching, which used the reactive ion etching (RIE) technique. To obtain a second mask pattern for Ti/Au contact padding, lift-off of Ti and Au was carried out after their deposition with a thermal evaporator (Auto 306 thermal evaporator; Edwards High Vacuum International, Wilmington, MA, USA). The cuneated PS electrode was first oxidized at 900°C in a dry O_2_ atmosphere to form an oxide layer while simultaneously expanding the PS. Subsequent BOE etching revealed this expanded PS, and the entire oxidation and etching process was repeated until the desired nanogap (NG) was achieved (size expansion technique). The oxidation time varied from 5 to 15 min in each cycle of oxidation, and etching depended on the thickness of the polysilicon layer. The final fabricated PSNG electrode, bonded to the microfluidic channel via plasma oxidation, is shown in Fig 2g. The microfluidic layer on the LOC was fabricated using an SU-8 soft-lithography microstructure mold (Fig 2h–2j). A mixture of Sylgard 184 Silicone Elastomer Base and Sylgard 184 Silicone Elastomer Curing Agent was poured onto the SU-8 microstructure mold to form the PDMS polymer.

### Surface modification on PSNG

#### PSNG surface hydroxylation

**Fig 3:**
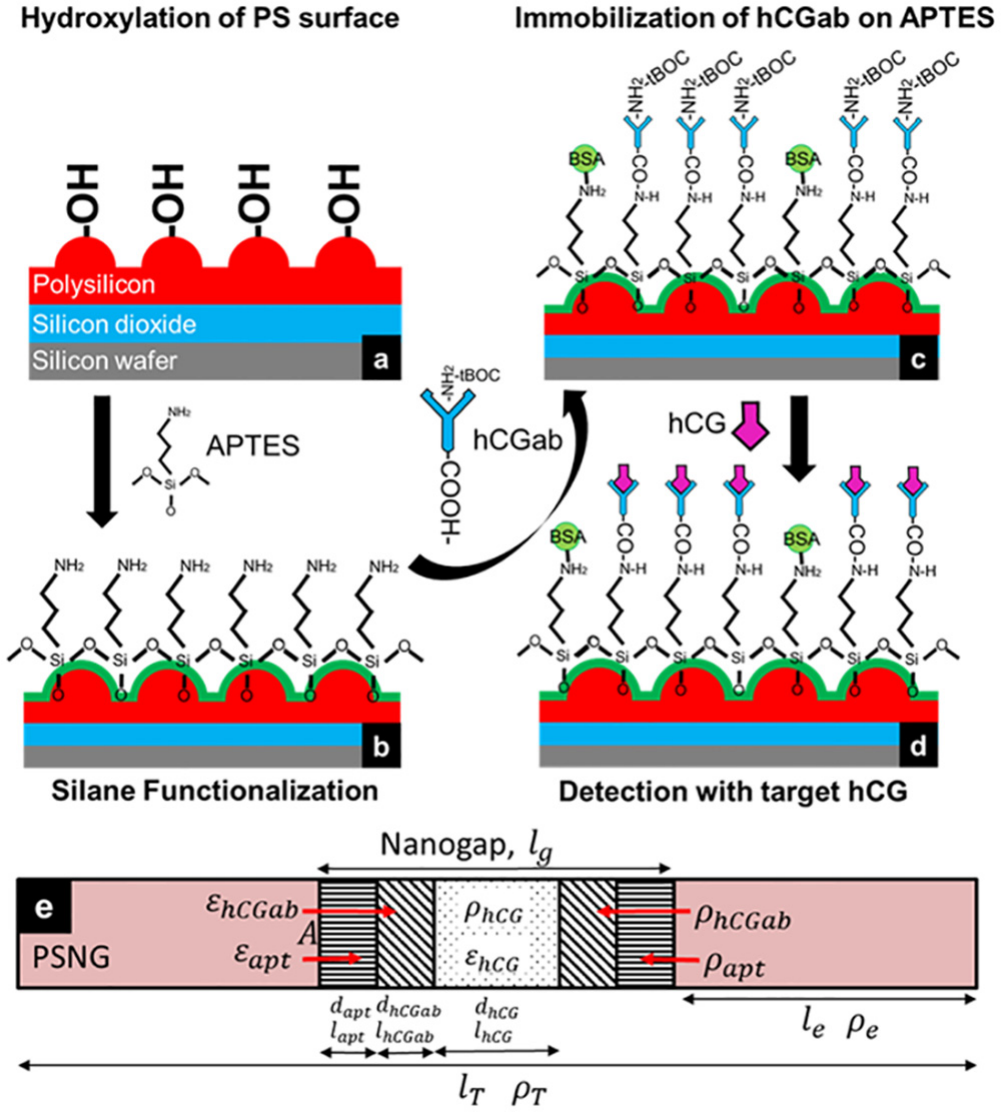
Schematic illustration of chemical functionalization on the PSNG surface. (a) Hydroxylation of the PSNG surface by Fenton reaction. (b) Application of the APTES cross-linker to establish amine terminated groups. (c) A prepared hCGab probe immobilized on the PSNG electrode. (d) hCG target binding with urine or reference samples. Signal transduction was analyzed in AC and DC measurements.

Because the PSNG structure is a non-biocompatible surface, it had to be modified to exhibit adhesion properties with biomolecules. The functionalization procedure is schematically shown in Fig 3. First, 10 ml of 0.1 M H_2_SO_4_ and 12.5 ml of H_2_O_2_ were diluted in water to a total volume of 100 ml. Then, 40 mg of EDTA were added, followed by 1.2 g of FeSO_4_ incrementally added to the solution, which reacted and heated up during the addition process. When the solution became bubbly and brown, the PSNG electrode was immersed for 30 min for hydroxylation to occur, which is known as a Fenton reaction. This process produces a–OH terminated group onto PSNG (Fig 3a).

#### hCGab immobilization on APTES

The amine-terminated PS surface was prepared by applying 1 μL of 10% APTES in water onto the PSNG electrode to establish a uniform layered surface (Fig 3b). The electrode was then incubated for 2 h and rinsed with ethanol to remove any unreacted APTES. hCGab (1 mg) was mixed with 1 ml DI water, 0.1 g of NaHCO_3_, the previously prepared mixture of 0.05 g tBOC and 1 ml chloroform. Then, 25 mM of EDAC and 50 mM of NHS were prepared in 0.01 M PBS solution. First, EDAC was added to the Ab solution and incubated for 20 min and then incubated in NHS for 10 min. Then, 1 μL of hCGab (8.6 mg/ml) solution was applied onto the PSNG electrode and incubated for 3 h at room temperature and humidity of 85.5% (Fig 3c). Subsequently, the remaining free surface amine groups on the PSNG electrode after Ab immobilization were blocked with 5% BSA in PBS for 10 min to avoid non-specific binding of hCG on the electrode. Then, the–NH_2_ protected by tBOC was removed by immersing the electrode in 10 mM TFA solution prior to detection with the hCG target [48].

#### hCG antigen binding

Clinical urine samples and hCG standard control solutions were reacted with the surface-modified PSNG electrode to analyze the signal transduction characteristics. Clinical samples consisting of pregnant and non-pregnant women’s urine were first analyzed in a clinical laboratory to estimate the hCG concentration in the urine and the presence of impurities. Furthermore, hCG standard control samples of 0, 8, 16, 40, 100, and 240 mIU/ml from the ELISA Kit were also applied to the PSNG electrode as a reference to validate the immunosensor. Before electrical characterization was conducted, 1 μL of hCG sample concentration was applied to the PSNG electrode and incubated for 3 h at room temperature and 85.5% humidity (Fig 3d).

### PSNG Electrode Characterization

#### Optical and morphological characterization

Inspections of the PSNG during fabrication were performed using high-power microscopy (OLYMPUS-BX51) to make sure no contaminants were present. The fabricated nanogaps were also inspected and their gap size measured using scanning electron microscopy (SEM, JEOL JSM-6460LA). Chemical functionalization and Ag-Ab surface modification on 1 x 1 cm PS surface samples were inspected using field emission scanning electron microscopy (FESEM, Zeiss Ultra55) and Fourier transform infrared spectroscopy (FTIR, Spectrum 65 Perkin-Elmer). Then, for surface roughness and grain size measurement of PS, atomic force microscopy (AFM) (SPA400-SPI3800, Seiko Instruments Inc., Japan) was used. Furthermore, the luminescence properties of APTES were studied using photoluminescence (PL) (Horiba Fluorolog-3 for PL spectroscopy, HORIBA Jobin Yvon Inc., USA). The thin-film layer thickness and uniformity were observed using a Filmetrics F20-UV spectrometer.

#### Electrical characterization

Electrical measurements were conducted to analyze signals transduced by the Ag-Ab binding reaction from the PSNG electrode, which were probed via the Au contact pad on the LOC (Fig A in S1 File). AC measurements were conducted using an impedance spectrometer (Alpha-A High Performance Frequency Analyzer, Novocontrol Technologies, Hundsangen, Germany). A two-wire probe station was used with the impedance spectrometer to measure capacitance (C), permittivity (ε), loss tangent (tan δ) and conductivity (σ). Measurement was started at 0.1 V with a scaling factor of 1.4 and a frequency range of (1 x 10^0^ to 1 x 10^6^ Hz). The loss tangent (tan δ) value was continuously monitored during capacitance measurement to ensure it stayed below 100, indicating that the immunosensor was behaving as a capacitor. A Kiethley 6487 Picoammeter was used to measure current-voltage DC characteristics of the PSNG electrode, from which amperometric characteristics and sensitivity of the immunosensor could be analyzed. At each step during hCG detection, the PSNG electrodes were washed with DI water to ensure that measurement data were valid.

## Results and Discussion

Several different sensing methods have been proposed to detect and discriminate hCG for pregnancy tests with various detection limits. Factors needed for a good sensor, such as sensitivity, selectivity, stability, reproducibility and simplicity, are critical and are achieved in the present study. Using the size expansion technique, a small nanogap size of 25 nm was achieved. Analyses were also conducted on the different gap sizes to measure their resistivity and the benefits of the small gap size. Analyses were performed using clinical urine samples and were correlated with hCG standard control samples for stability and selectivity. In addition, a single injection option facilitates triplicate data analyses, and the sensing surface can be regenerated.

### Morphological analysis of the PSNG

#### SEM, FESEM, FTIR and AFM analyses

**Fig 4:**
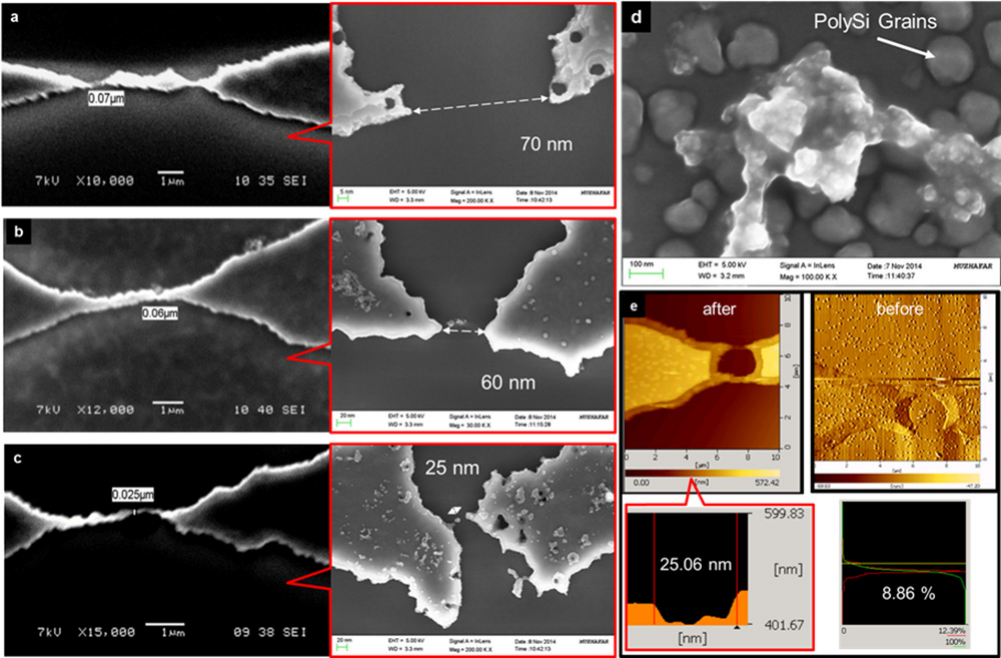
SEM, FESEM and AFM morphological analyses of the PSNG electrode. (a) SEM and FESEM (red box) image of 70 nm gap. (b) SEM and FESEM (red box) image of 60 nm gap (c) SEM and FESEM (red box) image of 25 nm gap (d) FESEM image of hCGab/hCG binding on PSNG electrode surface. (e) AFM images of electrode surface roughness before size expansion (right) and after size expansion (left); a final thickness of less than 100 nm and a bearing ratio of 6.86% were achieved.

SEM analysis of the fabricated PSNG electrodes showed that the smallest gap obtained was 25 nm from the size expansion process (Fig 4a–4c). Both SEM and FESEM (in red boxes) images were acquired to determine the gap sizes. Because the conventional lithography method was used, uneven nanogap geometrical shapes were the outcome. However, the reproducibility of the desired nanogaps is higher, at 49%, with nanogaps fabricated on a wafer, making this a great success (Fig B in S1 File). Surface modification and Ag-Ab binding on PSNG were examined under FTIR and FESEM. It was clearly observed that binding occurs on PS grains, which proves that good surface roughness is important for Ag-Ab attachments (Fig 4d). This finding was further supported by FTIR spectrometry, which could analyze PSNG/APTES, PSNG/APTES/hCGab and PSNG/APTES/hCGab/hCG complexes (Fig C in S1 File). The peaks of C-O stretching and N-H bending were observed at 1049–1201 cm^-1^ and 1641–1676 cm^-1^, respectively, demonstrating low transmittance peaks after hCG target binding. Therefore, this spectrum confirms the presence of an additional layer over hCGab. The PSNG surface roughness properties were examined using AFM, as shown in Fig 4e, which showed an overall surface roughness (S_a_) of 27.30 nm at a peak-valley (P-V) of 1.03 μm and an RMS value of 44.53 nm. However, with a small scan area, the surface roughness at the middle of the gap was S_a_ = 6.68 nm, and the P-V was 161.20 nm. This indicates that the fabricated PSNG structure exhibits a good surface-to-volume ratio that enhances the biocompatibility of the immunosensor [49].

#### Fluorometry analysis of polysilicon

**Fig 5:**
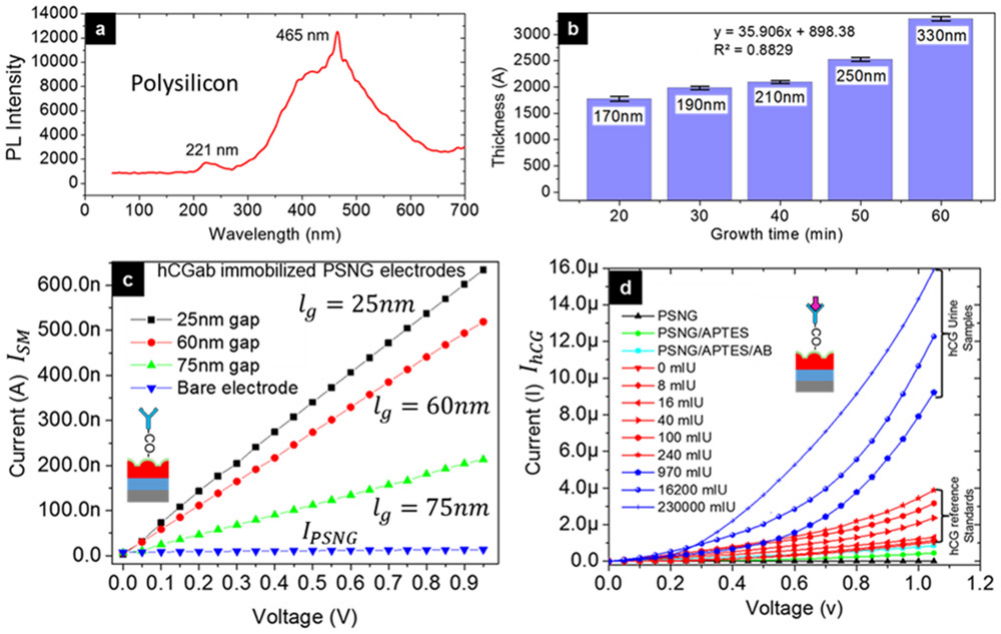
Morphological analysis and electrical characterization. (a) Polysilicon crystalline quality observed at UV and VIS regions from fluorometry analysis. (b) Step-by-step oxide growth for size expansion to achieve the nanogap-sized electrode. (c) Effect of different gap sizes on current transduction for APTES/AB immobilization. (d) DC amperometric detection of urine samples and hCG standard reference samples.

The photoluminescence properties of PS were examined using bare PS wafers via PL spectroscopy, with excitation from a 325 nm xenon lamp at both visible (VIS) and ultraviolet (UV) regions. Fig 5a shows the PL spectra of PS, which shows emission peaks at 221 and 465 nm. It is known that the emission band in the UV region is caused by a collisional recombination process and that the emission band in the VIS region is caused by electron-hole generation, depending on ionized vacancies. Therefore, the luminescence properties at both the UV and VIS regions reveal that the crystallinity of PS is not affected by a reduction in the annealing process [50].

#### Size expansion technique by oxide growth

The size expansion technique was used to reduce the gap size of PS. The initial patterned design on the silicon substrate had a 1 μm gap. During thermal oxidation, the patterned PS structure expanded as oxygen was being supplied (Fig 5b). After oxidation, the samples were immersed in BOE solution to remove excess oxide, leaving only the expanded underlying PS structure. Oxidation time reduced from 60 to 20 min (Fig 4a–4c) as the gap size reduced. The film thickness during each oxidation process was measured using Filmetrics Spectrometer F20-UV. Regression analysis of the oxidation process showed a linear regression, R^2^, of 0.8829 and linearity, y = 35.906x + 898.38, was achieved, where *x* is growth time and *y* is the film thickness. With the narrow nanogap obtained on our sensor, the effect of electrode polarization could be reduced, thus resulting in a negligible noise effect [37,51].

### Effect of gap size on PSNG sensitivity

The DC electrical characteristics of the PSNG electrode were examined during chemical functionalization and attachment of APTES and hCGab on the fabricated PSNG. Fig 5c illustrates the investigation of l_g_ = 75, 60 and 25 nm gap sizes, which were applied with 1 μL of prepared 8.6 mg/ml hCGab on APTES-modified PSNG. The graph shows that the resistance drops gradually when the gap size decreases from 75 to 60 to 25 nm, and the measured resistances were 65.7, 55.7, and 22.6 μΩ, respectively, as proven by Eqs 1 and 2. Therefore, to enhance the sensitivity of the electrode, a gap size of lg = 25 nm was selected for hCG measurement because it exhibits low resistance (S1 File).

$$PSNG\;current,\;I_{PSNG}=\frac{Av}{2\rho_{e}l_{e}+\;\rho_{air}l_{g}}$$ (1)

after surface modification with APTES and hCGab,

$$Surface\;modification\;current,\;I_{SM}=\frac{Av}{2(\rho_{e}l_{e}+\rho_{SM}l_{SM})}$$ (2)

It is also known that a small gap size, l_g_, could reduce the electrode polarization effect, which is a major source of error in nanogap electrodes. Therefore, it was suggested that l_g_ less than 100 nm could reduce the effect because the accumulation of ions known as the Debye layer at the smallest gap showed negligible error. Hence, reduction by l_g_ causes the Debye layer to overlap, and the potential drop could be reduced [37,52].

### DC and AC measurements on PSNG electrodes—clinical sample analyses

#### Current-voltage characteristics

The prepared PSNG electrode functionalized with APTES followed by hCGab immobilization was tested with pregnant and non-pregnant women’s urine samples, followed by hCG standard control samples. Each modified layer on the PSNG surface exhibits ohmic characteristics that influence the electrode resistivity according to R_T_ = (ρ_TlT_)/A. A total of six samples were tested (n = 6) for hCG, with three samples each from pregnant and non-pregnant women. In the pregnant women’s urine samples, the gestational ages of pregnancy were 4, 9 and 12 weeks, measured as 970, 16,200 and 230,000 mIU/ml, respectively, as obtained from a clinical laboratory. These results clearly indicated that there were no non-specific interferences by albumin, epithelial or glucose in the samples (Table 2). Therefore, by testing with our PSNG immunosensor, it was observed that an increase in hCG concentrations caused a reduction in current with both urine and hCG standard control samples (Fig 5d).

**Table 2:** Clinical analyses of urine samples using quantitative radioimmunoassays (RIA).

	Pregnant women			Non-pregnant women		
Sample	1	2	3	1	2	3
Gestational age	4 weeks	9 weeks	12 weeks	-	-	-
Albumin	Nd	Nd	Nd	Nd	Nd	Nd
Epithelial	Nd	Nd	Nd	Nd	Nd	Nd
Glucose level	Nd	Nd	Nd	Nd	Nd	Nd
Blood	No	No	No	No	No	No
hCG (mIU/ml)	970 ± 3.1	16200 ± 2.4	230000 ± 1.7	Nil	Nil	Nil

Note: Nd = Not detected.

$$hCG\;Current,I_{hCG}=\frac{Av}{2(\rho_{e}l_{e}+\rho_{SM}l_{SM})+\rho_{hCG}l_{hCG}}$$ (3)

Therefore, changes in hCG current, ΔI_hCG_, were due to the changes in resistivity of the hCG concentration, Δρ_hCG_, as proven in Eq 3, while other parameters remained constant (S1 File). It can thus be inferred that there is a binding event between hCGab and hCG on the surface of the PSNG electrode, which can transduce current based on hCG concentration [53].

#### Capacitance-frequency characteristics

During AC detection, each modification layer on the PSNG surface was signified as a multilayer dielectric material, and both PSNG electrodes were signified as parallel plate capacitors, thus behaving as a capacitive immunosensor based on capacitance, C = ɛ_r_ɛ_o_A/d. This distinctive property of the PSNG electrode was used in this study and was clearly reflected in the results obtained from capacitance, permittivity (Fig D in S1 File) and conductivity (Fig E in S1 File) signals upon hCG target binding on the electrodes. The capacitance and permittivity properties of PSNG increased when urine samples with higher hCG concentrations were applied, as shown in Eq 4. The changes in hCG concentration cause the change in hCG permittivity, Δɛ_hCG_, which eventually increases the hCG capacitance, C_hCG_.

$$hCG\;Capacitance,C_{hCG}=\frac{A\varepsilon_{e}\varepsilon_{SM}\varepsilon_{hCG}}{2\varepsilon_{hCG}(d_{e}\varepsilon_{SM}+d_{SM}\varepsilon_{e})+d_{hCG}\varepsilon_{e}\varepsilon_{SM}}$$ (4)

Therefore, those samples in which hCG has reacted with hCGab, forming a peptide bond, correspond to an increment in C_hCG_ of the PSNG electrode observed in the range 80 to 100 nF with the pregnant women’s urine samples [54]. However, samples without hCG do not exhibit this characteristic and exhibit C_hCG_ in the range of 30 to 40 nF. The sensor ɛ_hCG_ values ranged from 160 F/m to 185 F/m for pregnant samples. In addition, the measured capacitance is not only due to the sample capacitance but also influenced by the sample conductance, as shown in Eq 5. The sensor conductivity values ranged from 80 nS/cm to 100 nS/cm with increasing hCG levels in the pregnant urine samples. Lonappan et al. [55] and Sánchez et al. [56] have reported that an increment in hCG levels leads to an increased level of conductivity. Hence, in the sensor operation, it can be deduced that the measured capacitance values were due to the dielectric term and were also related to sample conductivity [57].

$$hCG\;Conductivity,\;\sigma =\frac{\varepsilon_{T}}{Z_{T}C_{hCG}}$$ (5)

### PSNG electrode analytical validation for specificity, sensitivity and stability

**Fig 6:**
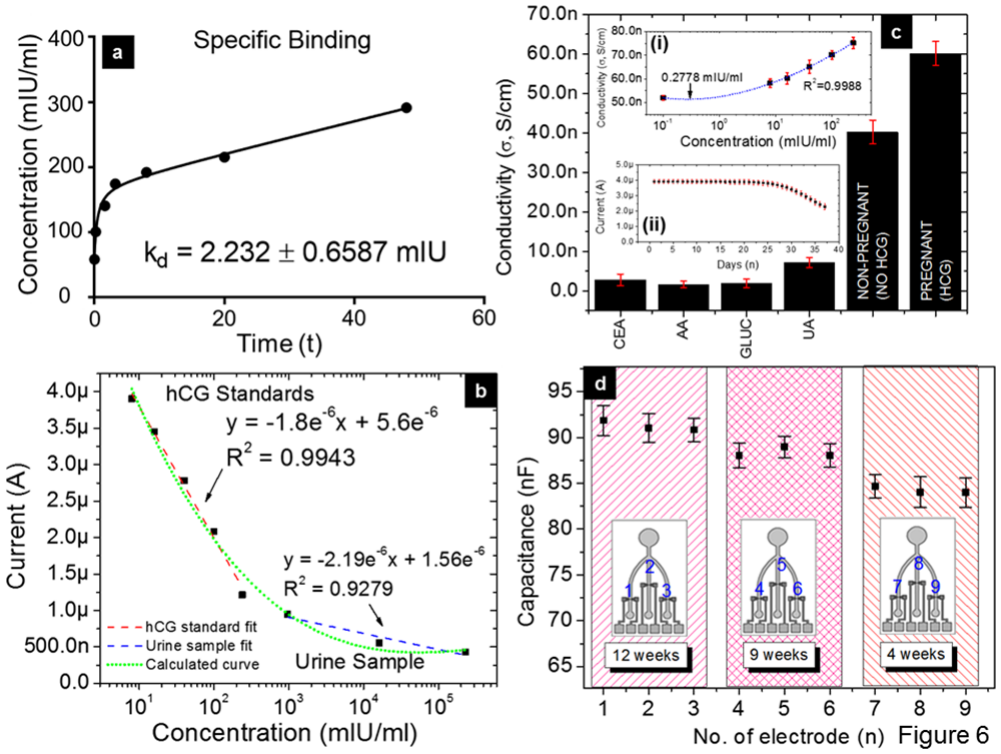
Analytical performance of the PSNG LOC immunosensor. (a) One-site specific binding to determine the protein dissociation constant K_d_. (b) Linearity fitting curves for hCG standards and urine samples. (c) Selectivity test with interfering species. CEA, AA, UA, glucose, non-pregnant and pregnant samples were used. The top inset is the AC conductivity test to determine the detection limit and the bottom inset shows that the immunosensor remains stable for 22 days. (d) Reproducibility test of signal transduction on PSNG electrodes with pregnant urine samples.

The obtained experimental results are highly valuable in calculating the performance of the sensor and its capabilities. In the present investigation, various analyses, such as Ag-Ab specific binding, sensitivity, selectivity, limit of detection (LOD), reproducibility and stability were carried out and are shown in Fig 6. During these tests, a non-specific blocking agent, BSA, was used to ensure that only hCG molecules bind to the hCGab probe. Therefore, one-site specific binding was analyzed, the equilibrium dissociation constant (K_d_) was calculated to be 2.23 ± 0.66 mIU, and the maximum number of binding sites per antigen would be B_max_ = 22.54 ± 1.46 mIU, as indicated in Fig 6a and Eq 6, where *x* is reaction time and *y* is hCG concentration.

$$y=\;\frac{22.54x}{2.232+x}$$ (6)

These results indicate a good affinity for hCG detection and the importance of sensitive PSNG electrodes in biosensing [58–60]. When a wide range of hCG concentrations were tested, sensitivity analysis determined the sensor’s ability to transduce significant currents. The linearity achieved for hCG standards was

$$I_{hCG(standard)}=\;-1.8e^{-6}[hCG]+5.6e^{-6}$$ (7)

with a correlation coefficient R^2^ = 0.9943, while for urine samples it was

$$I_{hCG(urine)}=\;-2.19e^{-6}[hCG]+1.56e^{-6}$$ (8)

with R^2^ = 0.9279 (Fig 6b). Therefore, our sensor exhibits a sensitivity of 835.88 μA mIU^-1^ ml^-2^ cm^-2^ at the active sensing area, with ‘A’ of 0.00262 cm^2^ and a gradient of 2.19 μA mIU^-1^ ml^-2^, as calculated from Eq 9. The proposed sensitivity is higher than the reported values of 0.255 μA mIU^-1^ ml^-2^ by Tan et al. [26] and 0.1032 μA mIU^-1^ ml^-2^ by Li et al. [27]. Here, the gradients of the urine sample plots are larger than standard reference samples, which could be due to a non-linear concentration range in the urine samples. Therefore, the gradients indicate normal behavior of urine samples in that they do not contain only hCG, unlike the ideal reference hCG samples. This proves that, even though our immunosensor is highly selective, the linearity depends on the type of sample. Therefore, this sensor could also measure hCG that exists in a different medium. Because the hCG concentration detected in urine samples is larger than that in the hCG standards, linearity proves that both slopes decrease as the concentration increases. This result has been further confirmed by plotting a curve that theoretically fits both the urine and standard samples.

$$Sensitivity=\frac{Slope\;of\;calibration\;plot,\;m\;(\mu A\;mM^{-1})}{Active\;Surface\;Area,\;A\;(cm^{2})}$$ (9)

Selectivity tests were carried out by using interfering species, such as CEA, AA, UA and glucose (GLUC), with 8 mIU/ml of hCG (Fig 6c). It can be observed that the response of interfering species on our PSNG electrode is less than 10 nS/cm at the lowest concentration of hCG. In addition, the LOD of the sensor was measured at 0.28 mIU/ml (27.78 pg/ml) at 3σ from PSNG electrode conductivity at a frequency of 1 MHz. To test this, a smaller concentration of the hCG target (0.1 mIU/ml) was prepared and its minimum conductivity saturation point was observed at 5.13 nS/cm, as shown in the top inset of (Fig 6c(i)). Hence, our LOD is proven to be considerably better than that of the nanoporous gold and graphene sensor reported by Li et al., at 0.34 mIU/ml [14] and polyethylene glycol microbeads reported by Zhu et al at 0.38 mIU/mL [61], as shown in Table 3. The Fig 6c(ii) inset shows that the stability of the present LOC decreases to 98.5% after 22 days of testing with 8 mIU/ml of hCG. This is acceptable for a sensor and comparable with the 7.1% decrease after 1 month using a MWCNT modified immunoassay, as reported by Na li et al. [27], and a 14% decrease after 3 weeks using a nanoporous gold and graphene sensor, as reported by Ru Li et al. [14].

**Table 3:** Comparison of different hCG immunosensors.

Electrode Matrix	Label	Linear Range	Detection Limit	Reference
PSNG/APTES/ hCGab/hCG	No	8–230,000 mIU/mL	0.28 mIU/mL	This work
*GCE^a^/GS^b^/NPG^c^/ hCGab/BSA/hCG	No	500–40,000 mIU/mL	0.34 mIU/mL	2011 [14]
*PEG^d^ microbeads/ hCGab/hCG	No	0.625–40 mIU/ml	0.38 mIU/mL	2015 [61]
Epitaxial graphene/ APTES/ hCGab/hCG	No	620–5620 mIU/mL	3.1 mIU/mL	2014 [13]
*GCE/ Pt-Au^e^ alloy nanotube array/ hCGab/hCG	No	25 to 400 mIU/mL	12 mIU/mL	2010 [15]
*Graphene-based CRET^f^ immunoassay	Yes	0.1–10 mIU/mL	0.06 mIU/mL	2014 [65]

*Note:

^a^Glassy Carbon Electrode,

^b^Graphene Sheets,

^c^Nanoporous Gold,

^d^Polyethylene Glycol,

^e^Platinum-Gold,

^f^Chemiluminescence Resonance Energy Transfer.

Reproducibility between LOCs was also tested to ensure intersensor signal accuracy. Fig 6d shows that there is little variation in capacitance with measured values of replica samples. However, it does not affect the electrode sensitivity, in which the relative standard deviations (RSDs) of 4.3, 3.3 and 4.9% changes in capacitance at 4, 9 and 12 weeks of pregnancy, respectively, indicate good reproducibility. This could be because the surface and geometrical factor of the PSNG electrode provide a good platform for biosensing [62]. Furthermore, for regeneration purposes, the PSNG sensor may also be reusable after washing it in urea to remove the bonded Ag target on immobilized APTES/hCGab [30]. This strategy enables the reuse of the sensing surface for multiple samples with tripartite sensing.

### Commercial potential of POC PSNG immunosensor

Our lab-on-chip immunosensor with electrical readouts offers several advantages over immunoassays due to its reduced cost, reduced form factor and ease of signal acquisition. The costs associated with laboratory equipment, chemicals and labeling procedures for hCG diagnosis via immunoassay methods could be reduced using POC immunosensors [63–65]. Because our POC PSNG is fabricated with multiple electrodes, it could facilitate multiplex detection from a single sample, which reduces cost and diagnosis time, as demonstrated in other sensing systems [33,66,67].

Recently, a study on the sensitivity and limitations of commercially available qualitative POC pregnancy test kits was reported by Greene et al. Two commonly used hCG POC tests, OSOM and QuickVue+, were examined, and the authors concluded that both devices have a detection range of 20–300 IU/ml with urine samples, which is insufficient for early pregnancy diagnosis. Furthermore, the mean gestational age for specimens corresponding to the false negative results was 4 weeks [68]. Siemens Immulite 1000 hCG Assay shows detection limit at 2.0 mIU/mL [69]. With our method, comparable sensitivity and specificity could be achieved from electrical transductions of the binding activity on PSNG, as shown in Table 4. Moreover, the gap size of 25 nm on the proposed lab-on-chip sensor is minimal compared to other commonly available lab-on-chip systems. In addition, the tripartite flow-channel minimizes the sample usage with uniform flow for parallel analyses. Overall, the PSNG electrode immunosensor used in the current study exhibited higher performance and a more facile fabrication process than other available systems, and the sensor can be readily fit with commercially available detection systems.

**Table 4:** Comparisons with commercially available POC pregnancy test kits.

POC devices	Analysis Method	Linear Range	Detection Limit	Reference
PSNG/APTES/ hCGab/hCG	Quantitative	8–230,000 mIU/mL	0.28 mIU/mL	This work
Siemens Immulite 1000 hCG Assay	Qualitative	2–3500 mIU/mL	2.0 mIU/mL	2013 [69]
QuickVue+	Qualitative	20–300 mIU/mL	>20 mIU/mL	2013 [68]
OSOM	Qualitative	20–300 mIU/mL	>20 mIU/mL	2013 [68]

## Conclusion

The human chorionic gonadotrophin (hCG) level in pregnant women is usually low during early pregnancy and increases drastically during the second trimester. As detection of hCG in the clinical urine sample is critical during pregnancy, we have demonstrated the detection of hCG with a surface functionalized PSNG electrode immunosensor fabricated by a conventional lithography and size expansion technique. Our lab-on-chip sensor exhibited good stability, high sensitivity (835.88 μA mIU^-1^ ml^-2^ cm^-2^), good reproducibility, high selectivity and a low detection limit (0.28 mIU/ml, or 27.78 pg/ml). Due to the narrow nanogap (25 nm) fabricated on this immunosensor, it shows high performance with a good signal-to-noise ratio, and it can be applied for multiplexed POC diagnosis, further reducing expensive equipment usage. Moreover, the dielectric analyses on the PSNG electrodes with clinical human urine samples provide reliable measurements and stability against regeneration reagents, which favors multiple sample analyses on the tripartite channel with the option of parallel runs.

## Supporting Information

S1 File This file contains. **Fig A.** (a) Electrical characterization setup for AC measurements using a frequency analyzer and (b) DC measurements using the Kiethley 6487 Picoammeter. **Fig B.** Reproducibility distribution of 25 nm gap on a single wafer per lithography. Of the fabricated electrodes, 49% have a gap sized of 25 nm. **Fig C.** FTIR spectrometry analyzed with PSNG/APTES, PSNG/APTES/hCGab and PSNG/APTES/hCGab/hCG samples. **Fig D.** AC capacitance and permittivity (inset) measurements for pregnant and non-pregnant women’s urine. **Fig E.** Conductivity measurements for pregnant and non-pregnant women’s urine.
(DOCX)

## References

[1] Liu J, Huang X, Cui M-L, Lin L, Zhang L, Zheng Z-Y, et al Determination of trace human chorionic gonadotropin by using multiwall carbon nanotubes as phosphorescence labeling reagent. Anal Biochem. Elsevier Inc.; 2012;431: 19–29. 10.1016/j.ab.2012.08.007 22906688

[2] Cole L a. New discoveries on the biology and detection of human chorionic gonadotropin. Reprod Biol Endocrinol. 2009;7: 8 10.1186/1477-7827-7-8 19171054 PMC2649930

[3] Cole LA. HCG, the wonder of today’s science. Reprod Biol Endocrinol. 2012;10. 10.1186/1477-7827-10-24 PMC3351023 22455390

[4] Perumal V, Hashim U. Advances in biosensors: principle, architecture and applications. J Appl Biomed. Korea Institute of Oriental Medicine; 2014;12: 1–15. 10.1016/j.jab.2013.02.001

[5] Gopinath SCB, Tang TH, Citartan M, Chen Y, Lakshmipriya T. Current aspects in immunosensors. Biosens Bioelectron. Elsevier; 2014;57: 292–302. 10.1016/j.bios.2014.02.029 24607580

[6] Piliarik M, Vaisocherová H, Homola J. A new surface plasmon resonance sensor for high-throughput screening applications. Biosens Bioelectron. 2005;20: 2104–2110. 10.1016/j.bios.2004.09.025 15741081

[7] Yang L, Zhao H, Fan S, Deng S, Lv Q, Lin J, et al Label-free electrochemical immunosensor based on gold-silicon carbide nanocomposites for sensitive detection of human chorionic gonadotrophin. Biosens Bioelectron. 2014;57: 199–206. 10.1016/j.bios.2014.02.019 24583692

[8] Liu D, Wu F, Zhou C, Shen H, Yuan H, Du Z, et al Multiplexed immunoassay biosensor for the detection of serum biomarkers—β-HCG and AFP of Down Syndrome based on photoluminescent water-soluble CdSe/ZnS quantum dots. Sensors Actuators B Chem. 2013;186: 235–243.

[9] Davison CM, Kaplan RM, Wenig LN, Burmeister D. Qualitative beta-hCG urine assays may be misleading in the presence of molar pregnancy: a case report. J Emerg Med. 2004;27: 43–7. 10.1016/j.jemermed.2004.02.009 15219303

[10] Yang G, Yang X, Yang C, Yang Y. A reagentless amperometric immunosensor for human chorionic gonadotrophin based on a gold nanotube arrays electrode. Colloids Surfaces A Physicochem Eng Asp. Elsevier B.V.; 2011;389: 195–200. 10.1016/j.colsurfa.2011.08.027

[11] Wei Q, Li R, Du B, Wu D, Han Y, Cai Y, et al Multifunctional mesoporous silica nanoparticles as sensitive labels for immunoassay of human chorionic gonadotropin. Sensors Actuators B Chem. 2011;153: 256–260. 10.1016/j.snb.2010.09.073

[12] Yang H, Yuan R, Chai Y, Su H, Zhuo Y, Jiang W, et al Electrochemical immunosensor for human chorionic gonadotropin based on horseradish peroxidase–functionalized Prussian blue–carbon nanotubes/gold nanocomposites as labels for signal amplification. Electrochim Acta. Elsevier Ltd; 2011;56: 1973–1980.

[13] Teixeira S, Burwell G, Castaing a., Gonzalez D, Conlan RS, Guy OJ. Epitaxial graphene immunosensor for human chorionic gonadotropin. Sensors Actuators B Chem. Elsevier B.V.; 2014;190: 723–729. 10.1016/j.snb.2013.09.019

[14] Li R, Wu D, Li H, Xu C, Wang H, Zhao Y, et al Label-free amperometric immunosensor for the detection of human serum chorionic gonadotropin based on nanoporous gold and graphene. Anal Biochem. 2011;414: 196–201. 10.1016/j.ab.2011.03.019 21435334

[15] Tao M, Li X, Wu Z, Wang M, Hua M, Yang Y. The preparation of label-free electrochemical immunosensor based on the Pt-Au alloy nanotube array for detection of human chorionic gonadotrophin. Clin Chim Acta. Elsevier B.V.; 2011;412: 550–5. 10.1016/j.cca.2010.12.004 21146510

[16] Chai R, Yuan R, Chai Y, Ou C, Cao S, Li X. Amperometric immunosensors based on layer-by-layer assembly of gold nanoparticles and methylene blue on thiourea modified glassy carbon electrode for determination of human chorionic gonadotrophin. Talanta. 2008;74: 1330–6. 10.1016/j.talanta.2007.08.046 18371786

[17] Xuan Viet N, Chikae M, Ukita Y, Maehashi K, Matsumoto K, Tamiya E, et al Gold-linked electrochemical immunoassay on single-walled carbon nanotube for highly sensitive detection of human chorionic gonadotropin hormone. Biosens Bioelectron. Elsevier; 2013;42: 592–7. 10.1016/j.bios.2012.11.017 23261694

[18] Severs AH, Schasfoort RBM. Enhanced surface plasmon resonance inhibition test (ESPRIT) using latex particles. Biosens Bioelectron. 1993;8: 365–370.

[19] Kelly LS, Kozak M, Walker T, Pierce M, Puett D. Lectin immunoassays using antibody fragments to detect glycoforms of human chorionic gonadotropin secreted by choriocarcinoma cells. Anal Biochem. 2005;338: 253–62. 10.1016/j.ab.2004.12.011 15745745

[20] Lim T-KK, Matsunaga T. Construction of electrochemical flow immunoassay system using capillary columns and ferrocene conjugated immunoglobulin G for detection of human chorionic gonadotrophin. Biosens Bioelectron. 2001;16: 1063–1069. 10.1016/S0956-5663(01)00228-7 11679290

[21] Qin Q, Christiansen M, Lövgren T, Nørgaard-Pedersen B, Pettersson K. Dual-label time-resolved immunofluorometric assay for simultaneous determination of pregnancy-associated plasma protein A and free beta-subunit of human chorionic gonadotrophin. J Immunol Methods. 1997;205: 169–175. 9294599 10.1016/s0022-1759(97)00073-2

[22] Prasad P V., Chaube SK, Panchal M, Chaudhary R, Muralidhar K, Rohil V, et al Molecular dissection of an hCG-?? epitope using single-step solid phase radioimmunoassay. Clin Chim Acta. 2007;376: 52–59. 10.1016/j.cca.2006.07.013 16959230

[23] Yuhi T, Nagatani N, Endo T, Kerman K, Takata M, Konaka H, et al Gold nanoparticle based immunochromatography using a resin modified micropipette tip for rapid and simple detection of human chorionic gonadotropin hormone and prostate-specific antigen. Sci Technol Adv Mater. 2006;7: 276–281.

[24] Yuhi T, Nagatani N, Endo T, Kerman K, Takata M, Konaka H, et al Resin-based micropipette tip for immunochromatographic assays in urine samples. J Immunol Methods. 2006;312: 54–60. 10.1016/j.jim.2006.02.011 16624320

[25] Jiang Z-L, Zou M-J, Liang A-H. An immunonanogold resonance scattering spectral probe for rapid assay of human chorionic gonadotrophin. Clin Chim Acta. 2008;387: 24–30. 10.1016/j.cca.2007.08.017 17936256

[26] Tan F, Yan F, Ju H. Sensitive reagentless electrochemical immunosensor based on an ormosil sol-gel membrane for human chorionic gonadotrophin. Biosens Bioelectron. 2007;22: 2945–51. 10.1016/j.bios.2006.12.010 17223029

[27] Li N, Yuan R, Chai Y, Chen S, An H. Sensitive immunoassay of human chorionic gonadotrophin based on multi-walled carbon nanotube-chitosan matrix. Bioprocess Biosyst Eng. 2008;31: 551–8. 10.1007/s00449-008-0201-0 18324418

[28] Yang H, Yuan R, Chai Y, Zhuo Y. Electrochemically deposited nanocomposite of chitosan and carbon nanotubes for detection of human chorionic gonadotrophin. Colloids Surf B Biointerfaces. Elsevier B.V.; 2011;82: 463–9. 10.1016/j.colsurfb.2010.10.003 21030221

[29] Chen J, Yan F, Dai Z, Ju H. Reagentless amperometric immunosensor for human chorionic gonadotrophin based on direct electrochemistry of horseradish peroxidase. Biosens Bioelectron. 2005;21: 330–6. 10.1016/j.bios.2004.10.023 16023960

[30] Liao J-Y. Detection of human chorionic gonadotrophin hormone using a label-free epoxysilane-modified capacitive immunosensor. Appl Microbiol Biotechnol. 2007;74: 1385–91. 10.1007/s00253-006-0778-7 17160390

[31] Truong LTN, Chikae M, Ukita Y, Takamura Y. Labelless impedance immunosensor based on polypyrrole-pyrolecarboxylic acid copolymer for hCG detection. Talanta. Elsevier B.V.; 2011;85: 2576–80. 10.1016/j.talanta.2011.08.018 21962685

[32] Perumal V, Hashim U, Gopinath SCB, Haarindraprasad R, Foo KL, Balakrishnan SR, et al “Spotted Nanoflowers”: Gold-seeded Zinc Oxide Nanohybrid for Selective Bio-capture. Sci Rep. Nature Publishing Group; 2015;5: 12231 10.1038/srep12231 PMC4503952 26178973

[33] Qureshi A, Niazi JH, Kallempudi S, Gurbuz Y. Label-free capacitive biosensor for sensitive detection of multiple biomarkers using gold interdigitated capacitor arrays. Biosens Bioelectron. Elsevier B.V.; 2010;25: 2318–23. 10.1016/j.bios.2010.03.018 20381333

[34] Hsu P-Y, Lin J-J, Wu Y-L, Hung W-C, Cullis AG. Ultra-sensitive polysilicon wire glucose sensor using a 3-aminopropyltriethoxysilane and polydimethylsiloxane-treated hydrophobic fumed silica nanoparticle mixture as the sensing membrane. Sensors Actuators B Chem. 2009;142: 273–279. 10.1016/j.snb.2009.08.003

[35] Wu Y-L, Lin J-J, Hsu P-Y, Hsu C-P. Highly sensitive polysilicon wire sensor for DNA detection using silica nanoparticles/γ-APTES nanocomposite for surface modification. Sensors Actuators B Chem. Elsevier B.V.; 2011;155: 709–715.

[36] Zhang G-J, Ning Y. Silicon nanowire biosensor and its applications in disease diagnostics: A review. Anal Chim Acta. Elsevier B.V.; 2012;749: 1–15. 10.1016/j.aca.2012.08.035 23036462

[37] Balakrishnan SR, Hashim U, Letchumanan GR, Kashif M, Ruslinda a. R, Liu WW, et al Development of Highly Sensitive Polysilicon Nanogap with APTES/GOx Based Lab-On-Chip Biosensor to Determine Low Levels of Salivary Glucose. Sensors Actuators A Phys. Elsevier B.V.; 2014;220: 101–111. 10.1016/j.sna.2014.09.027

[38] Rao BS, Hashim U, Dhahi T, Adam T. DI Water Electrical Characteristics Monitoring Using in House Fabricated Polysilicon Nanoelectrode Based Transducer. Int J Enhanc Res Sci Technol Eng. 2012;1: 1–6.

[39] Rao BS, Hashim U, Dhahi TS, Adam T. pH sensing using in house fabricated polysilicon nanoelectrode based transducer. Biomedical Engineering and Sciences (IECBES), 2012 IEEE EMBS Conference on. 2012. pp. 122–125. 10.1109/IECBES.2012.6498063

[40] Rao B, Hashim U. Pattern Transfer of 1μm Sized Microgap and Microbridge Electrode for Application in Biomedical Nano-Diagnostics. Adv Mater Res. 2014;925: 533–537.

[41] Rao BS, Hashim U. Microfluidic Photomask Design using CAD Software for Application in Lab-On-Chip Biomedical Nano Diagnostics. Adv Mater Res. 2013;795: 388–392.

[42] Rao BS, Hashim U. Nanoelectrode Chrome Photomask Design and Specification for Biosensor Fabrication. Adv Mater Res. 2013;795: 397–402. 10.4028/www.scientific.net/AMR.795.397

[43] Rao B, Nurfaiz M, Hashim U. Quantitative measurement of sugar concentration using in house fabricated microgap biosensor. 2013 IEEE Regional Symposium on Micro and Nanoelectronics (RSM). 2013. pp. 54–57.

[44] Rao B, Nurfaiz M, Hashim U. Photoresist microbridge pattern optimization at 1μm using conventional photolithography technique. 2013 IEEE Regional Symposium on Micro and Nanoelectronics (RSM). 2013. pp. 17–20.

[45] Rao BS, Hashim U, Adam T. Thin Film Thickness and Uniformity Measurement for Lab-on-Chip Based Nanoelectrode Biosensor Development. Adv Mater Res. 2014;832: 95–100. 10.4028/www.scientific.net/AMR.832.95

[46] Rao BS, Asri M. Conventional Photolithography and Process Optimization of Pattern-Size Expansion Technique for Nanogap Biosensor Fabrication. Adv Mater Res. 2014;832: 89–94. 10.4028/www.scientific.net/AMR.832.89

[47] Liu W-W, Hashim U, Rao S. Carbon nanotubes-based electrochemical biosensors. Biomedical Engineering and Sciences (IECBES), 2012 IEEE EMBS Conference on. 2012. pp. 392–397. 10.1109/IECBES.2012.6498144

[48] Gregor CR, Cerasoli E, Schouten J, Ravi J, Slootstra J, Horgan A, et al Antibody recognition of a human chorionic gonadotropin epitope (hCGbeta66-80) depends on local structure retained in the free peptide. J Biol Chem. 2011;286: 25016–26. 10.1074/jbc.M111.246637 21592960 PMC3137075

[49] Hsu C-W, Wang G-J. Highly sensitive glucose biosensor based on Au–Ni coaxial nanorod array having high aspect ratio. Biosens Bioelectron. Elsevier; 2014;56: 204–209. 10.1016/j.bios.2014.01.023 24495482

[50] Foo KL, Hashim U, Muhammad K, Voon CH. Sol—gel synthesized zinc oxide nanorods and their structural and optical investigation for optoelectronic application. 2014;9: 1–10. 10.1186/1556-276X-9-429 PMC4150024 25221458

[51] Mingqiang Yi Luke P. Lee K-HJ, Mingqiang Yi Luke P. Lee K-HJ, Yi M, Jeong K-H, Lee LP. Theoretical and experimental study towards a nanogap dielectric biosensor. Biosens Bioelectron. 2005;20 (2005): pp.1320–1326. 10.1016/j.bios.2004.05.003 15590285

[52] Oh S, Lee J, Jeong K, Lee L, SebaekOh Ki-Hun Jeong, and Luke P. Lee JSL. Minimization of electrode polarization effect by nanogap electrodes for biosensor applications. Micro Electro Mech …. 2003;52–55(2003: 52–55.

[53] Santandreu M, Alegret S, Fàbregas E. Determination of β-HCG using amperometric immunosensors based on a conducting immunocomposite. Anal Chim Acta. 1999;396: 181–188.

[54] Bourinbaiar AS, Powell JE, Stevens VC. The role of carboxy-terminal portion of beta subunit of human chorionic gonadotropin in human immunodeficiency virus infection. Life Sci. Elsevier; 1997;61: PL149–PL157. 10.1016/s0024-3205(97)00568-7 9307056

[55] Lonappan A, Rajasekaran C, Thomas V, Bindu G, Mathew KT. Determination of pregnancy using microwaves. Microw Opt Technol Lett. 2007;49: 786–788. 10.1002/mop.22279

[56] Sánchez S, Roldán M, Pérez S, Fàbregas E. Toward a fast, easy, and versatile immobilization of biomolecules into carbon nanotube/polysulfone-based biosensors for the detection of hCG hormone. Anal Chem. 2008;80: 6508–14. 10.1021/ac7025282 18662016

[57] Heidari M, Azimi P. Conductivity effect on the capacitance measurement of a parallel-plate capacitive sensor system. African Rev Phys. 2011;4.

[58] Pan C. Measuring dissociation rate constants of protein complexes through subunit exchange: Experimental design and theoretical modeling. PLoS One. 2011;6 10.1371/journal.pone.0028827 PMC3237551 22194924

[59] Docoslis A, Wu W, Giese RF, Van Oss CJ. Measurements of the kinetic constants of protein adsorption onto silica particles. Colloids Surfaces B Biointerfaces. 1999;13: 83–104. 10.1016/S0927-7765(98)00111-8

[60] Docoslis A, Rusinski L a., Giese RF, Van Oss CJ. Kinetics and interaction constants of protein adsorption onto mineral microparticles—Measurement of the constants at the onset of hysteresis. Colloids Surfaces B Biointerfaces. 2001;22: 267–283.

[61] Zhu Q, Trau D. Gel pad array chip for high throughput and multi-analyte microbead-based immunoassays. Biosens Bioelectron. Elsevier; 2015;66: 370–378. 10.1016/j.bios.2014.10.083 25463645

[62] Haarindraprasad R, Hashim U, Gopinath SCB, Kashif M, Veeradasan P, Balakrishnan SR, et al Low Temperature Annealed Zinc Oxide Nanostructured Thin Film-Based Transducers: Characterization for Sensing Applications. PLoS One. 2015;10: e0132755 10.1371/journal.pone.0132755 26167853 PMC4500498

[63] Kaushik A, Vasudev A, Arya SK, Pasha SK, Bhansali S. Recent advances in cortisol sensing technologies for point-of-care application. Biosens Bioelectron. Elsevier; 2014;53: 499–512. 10.1016/j.bios.2013.09.060 24212052

[64] Sharma MK, Agarwal GS, Rao VK, Upadhyay S, Rai GP, Vijayaraghavan R. Amperometric Biosensor for the Sensitive Detection of Plasmodium falciparum Histidine Rich Protein-2 Antigen. Sens Lett. 2011;9: 1363–1369. 10.1166/sl.2011.1681

[65] Lei J, Jing T, Zhou T, Zhou YY, Wu W, Mei S, et al A simple and sensitive immunoassay for the determination of human chorionic gonadotropin by graphene-based chemiluminescence resonance energy transfer. Biosens Bioelectron. Elsevier; 2014;54: 72–7. 10.1016/j.bios.2013.10.033 24252762

[66] Gopinath SCB, Awazu K, Tominaga J, Kumar PKR. Monitoring biomolecular interactions on a digital versatile disk: A BioDVD platform technology. ACS Nano. 2008;2: 1885–1895. 10.1021/nn800285p 19206429

[67] Gopinath SCB, Awazu K, Fujimaki M, Shimizu K. Evaluation of Anti-A / Udorn / 307 / 1972 Antibody Specificity to Influenza A / H3N2 Viruses Using an Evanescent-Field Coupled Waveguide-Mode Sensor. PLoS One. 2013;8: 20–21. 10.1371/journal.pone.0081396 PMC3858306 24339924

[68] Greene DN, Schmidt RL, Kamer SM, Grenache DG, Hoke C, Lorey TS. Limitations in qualitative point of care hCG tests for detecting early pregnancy. Clin Chim Acta. Elsevier B.V.; 2013;415: 317–21. 10.1016/j.cca.2012.10.053 23159297

[69] Cate FL, Moffett C, Gronowski AM, Grenache DG, Hartmann KE, Woodworth A. Analytical and clinical validation of the Immulite 1000 hCG assay for quantitative analysis in urine. Clin Chim Acta. Elsevier B.V.; 2013;421: 104–8. 10.1016/j.cca.2013.02.026 PMC3856699 23470427

