# Supplementary material for: A Point-of-Care Immunosensor for Human Chorionic Gonadotropin in Clinical Urine Samples Using a Cuneated Polysilicon Nanogap Lab-on-Chip
Source: PLoS One. 2015 Sep 14;10(9):e0137891. doi: 10.1371/journal.pone.0137891 (PMC4569379; doi:10.1371/journal.pone.0137891)
Supplement: S1 File — Fig A. (a) Electrical characterization setup for AC measurements using a frequency analyzer and (b) DC measurements using the Kiethley 6487 Picoammeter. Fig B. Reproducibility distribution of 25 nm gap on a single wafer per lithography. Of the fabricated electrodes, 49% have a gap sized of 25 nm. Fig C. FTIR spectrometry analyzed with PSNG/APTES, PSNG/APTES/hCGab and PSNG/APTES/hCGab/hCG samples. Fig D. AC capacitance and permittivity (inset) measurements for pregnant and non-pregnant women’s urine. Fig E. Conductivity measurements for pregnant and non-pregnant women’s urine. (DOCX) [file pone.0137891.s001.docx]

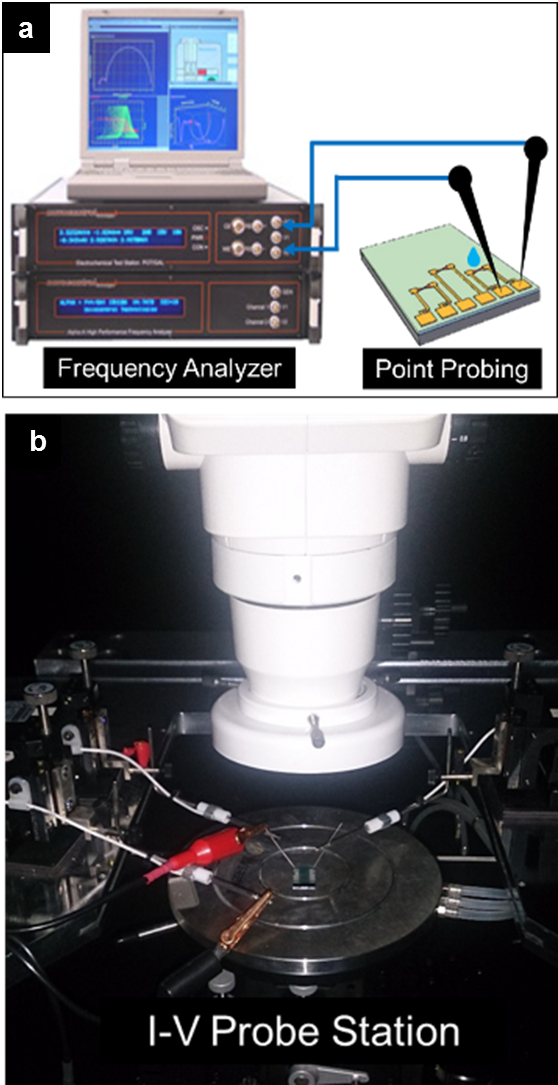


**Figure A.** (a) Electrical characterization setup for AC measurements using a frequency analyzer and (b) DC measurements using the Kiethley 6487 Picoammeter.


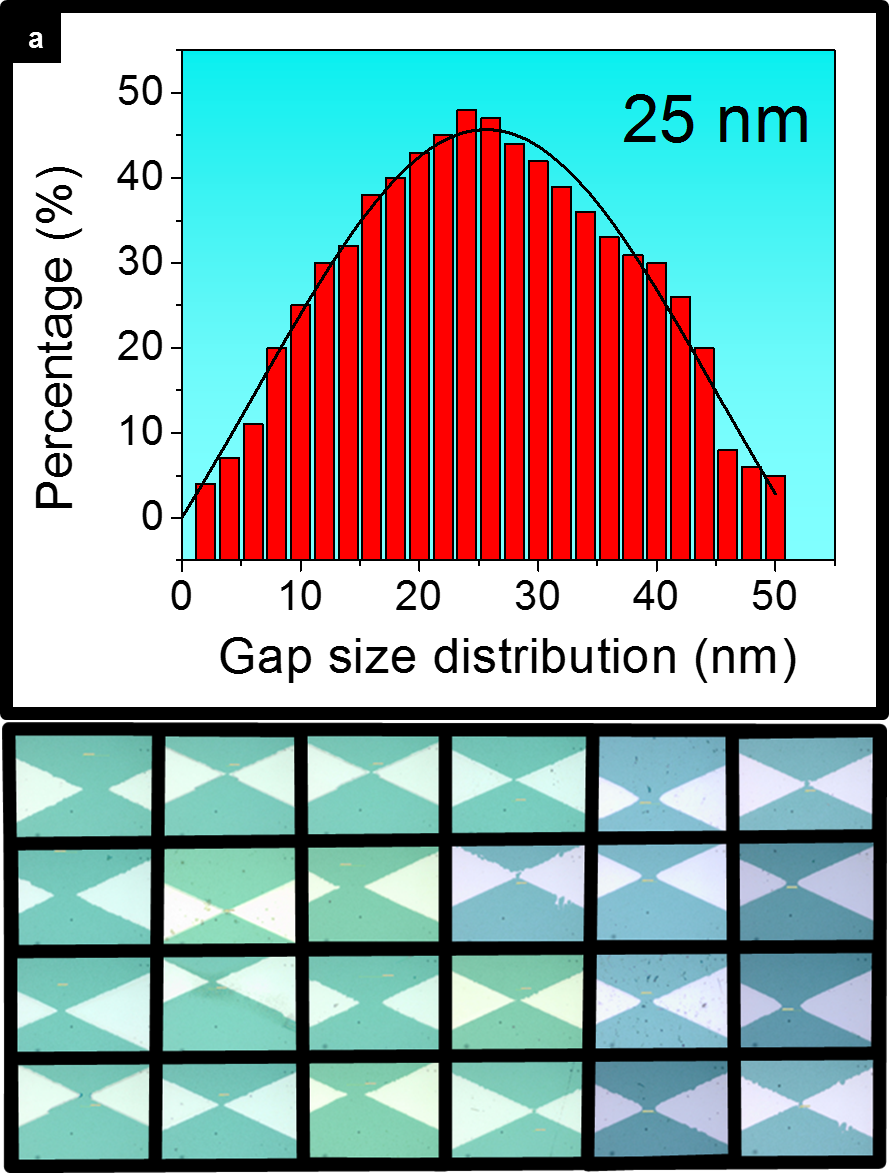


**Figure B.** Reproducibility distribution of 25 nm gap on a single wafer per lithography. Of the fabricated electrodes, 49% have a gap sized of 25 nm.

## FTIR analysis

Fig. S3 shows FTIR spectroscopy analysis with PSNG/APTES, PSNG/APTES/hCGab and PSNG/APTES/hCGab/hCG samples. Peaks of O-H stretch at 2938 and 3056 cm^-1^ shows the presence of intermolecular hydrogen bonding between APTES, hCGab and hCG. After hCGab and hCG is applied onto APTES coated PS substrate, the transmittance intensity drops rapidly due to the difference in light absorption which causes the peak. Hence, from peaks of C-O stretch and N-H bend observed at 1029-1201 cm^-1^ and 1641-1676 cm^-1^, it proves formation of peptide bond between hCGab and hCG during the incubation process. Overall, the intense peak after presence of additional hCG confirms the bonding on the hCGab.

**Figure C.** FTIR spectrometry analyzed with PSNG/APTES, PSNG/APTES/hCGab and PSNG/APTES/hCGab/hCG samples.

## Current-voltage characteristics on PSNG electrode

The current transduced by PSNG electrode involves the summation of total resistance, R_T_ that includes two parts of PSNG electrode resistance (R_e_), two parts of APTES resistance (R_apt_), two parts of hCGab resistance (R_ab_) and one part of hCG resistance (R_hcg_).

$$R_{T}=R_{e}+R_{apt}+R_{hCGab}+R_{hCG}$$

Since there is different resistive materials, the resistance were calculated based on each materials resistivity.

$$R_{T}=2\left( \frac{\rho_{e}l_{e}}{A}+\frac{\rho_{apt}l_{apt}}{A}+\frac{\rho_{hCGab}l_{hCGab}}{A} \right)+\frac{\rho_{hCG}l_{hCG}}{A}$$

Hence, adding R_T_ into current-voltage function,

$$I_{hCG}=\left( \frac{1}{R_{T}} \right)v$$

By letting,

$$\frac{\rho_{apt}l_{apt}}{A}+\frac{\rho_{hCGab}l_{hCGab}}{A}=\frac{\rho_{SM}l_{SM}}{A}$$

$$R_{T}=\frac{2\rho_{e}l_{e}}{A}+\frac{2\rho_{SM}l_{SM}}{A}+\frac{\rho_{hCG}l_{hCG}}{A}$$

$$\frac{1}{R_{T}}=\frac{A}{2\left( \rho_{e}l_{e}+\rho_{SM}l_{SM} \right)+\rho_{hCG}l_{hCG}}$$

Hence, current exhibition during hCG detection,

$$I_{hCG}=\frac{Av}{2\left( \rho_{e}l_{e}+\rho_{SM}l_{SM} \right)+\rho_{hCG}l_{hCG}}$$

current exhibition for APTES and hCGab surface modification,

$$I_{SM}=\frac{Av}{2\left( \rho_{e}l_{e}+\rho_{SM}l_{SM} \right)}$$

and current exhibition with bare PSNG electrode,

$$I_{PSNG}=\frac{Av}{2\left( \rho_{e}l_{e}+\rho_{air}l_{g} \right)}$$

Therefore, from here it can be deduced that the large difference in current for Pair and Psm was determined by the air and surface modification resistivity. Hence, in both condition, Pair and Psm were the contributing factor to Ipsng and Ism and the reason curve in Fig. 5c exhibits linear function. Whereas, in Fig. 5d, it is observed that during hCG detection the curve approaches non-linear function as the hCG concentration increases rapidly. This could be due to the addition of phcg factor during hCG current transduction, Ihcg in which increment in phcg was contributed by the increasing hCG concentration in the sample that causes the curve to grow exponentially.

## Capacitance-frequency characteristics on PSNG electrode

The PSNG electrode was examined in AC environment to analyse the dielectric properties with hCG detection. Hence, the electrode that is coated with multi layered surface modification caused the device to behave as multiple dielectric contained capacitive sensor as in Fig. S4. Therefore, the total capacitance, Ct was contributed by summation of serial arrangement of two layer of electrode capacitance (Ce), two layer of APTES capacitance (Capt), another two layer of hCGab immobilized capacitance (Chcgab) and one layer of hCG capacitance (Chcg) as following,

$$\frac{1}{C_{T}}=2\left( \frac{1}{C_{e}}+\frac{1}{C_{apt}}+\frac{1}{C_{hCGab}} \right)+\frac{1}{C_{hCG}}$$

from C=ɛA/d,

$$\frac{1}{C_{T}}=2\left( \frac{d_{e}}{{A\varepsilon}_{e}}+\frac{d_{apt}}{{A\varepsilon}_{apt}}+\frac{d_{hCGab}}{{A\varepsilon}_{hCGab}} \right)+\frac{d_{hCG}}{A\varepsilon_{hCG}}$$

By letting,

$$\frac{d_{apt}}{A\varepsilon_{apt}}+\frac{d_{hCGab}}{A\varepsilon_{hCGab}}=\frac{d_{SM}}{A\varepsilon_{SM}}$$

$$\frac{1}{C_{T}}=2\left( \frac{d_{e}}{{A\varepsilon}_{e}}+\frac{d_{SM}}{{A\varepsilon}_{SM}} \right)+\frac{d_{hCG}}{A\varepsilon_{hCG}}$$

Hence capacitance exhibited during hCG detection,

$$hCG Capacitance,C_{hCG}=C_{T}=\frac{A\varepsilon_{e}\varepsilon_{SM}\varepsilon_{hCG}}{2\varepsilon_{hCG}\left( d_{e}\varepsilon_{SM}+d_{SM}\varepsilon_{e} \right)+d_{hCG}\varepsilon_{e}\varepsilon_{SM}}$$

the equation above satisfies the curves in Fig. S3, that both permittivity and capacitance increases when hCG concentration increases.


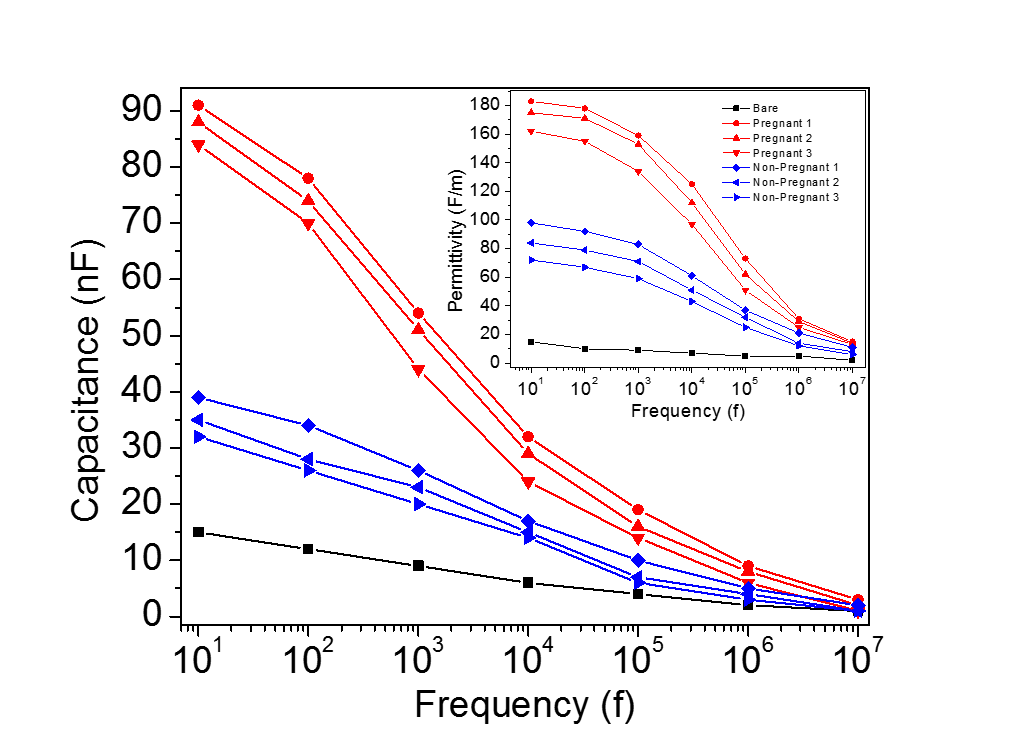


**Figure D.** AC capacitance and permittivity (inset) measurements for pregnant and non-pregnant women’s urine.

The conductivity of the PSNG electrode was also examined during AC dielectric measurement. Fig. S5 illustrates the conductivity of pregnant and non-pregnant women urine sample is inversely proportional with the capacitance. It is so because the liquid sample between parallel PSNG electrodes behaves not only as a dielectric material, but also as a conductive liquid which due to the Impedance, Z. The association between current, I, capacitance, C and conductivity, σ could be further derived as follows;

from,

$$Conductivity, \sigma=\frac{1}{Z}\left( \frac{d}{A} \right)$$

hence, from capacitance, C = (ɛA)/d,

$$Conductivity, \sigma=\frac{\varepsilon_{T}}{Z_{T}C_{hCG}}$$

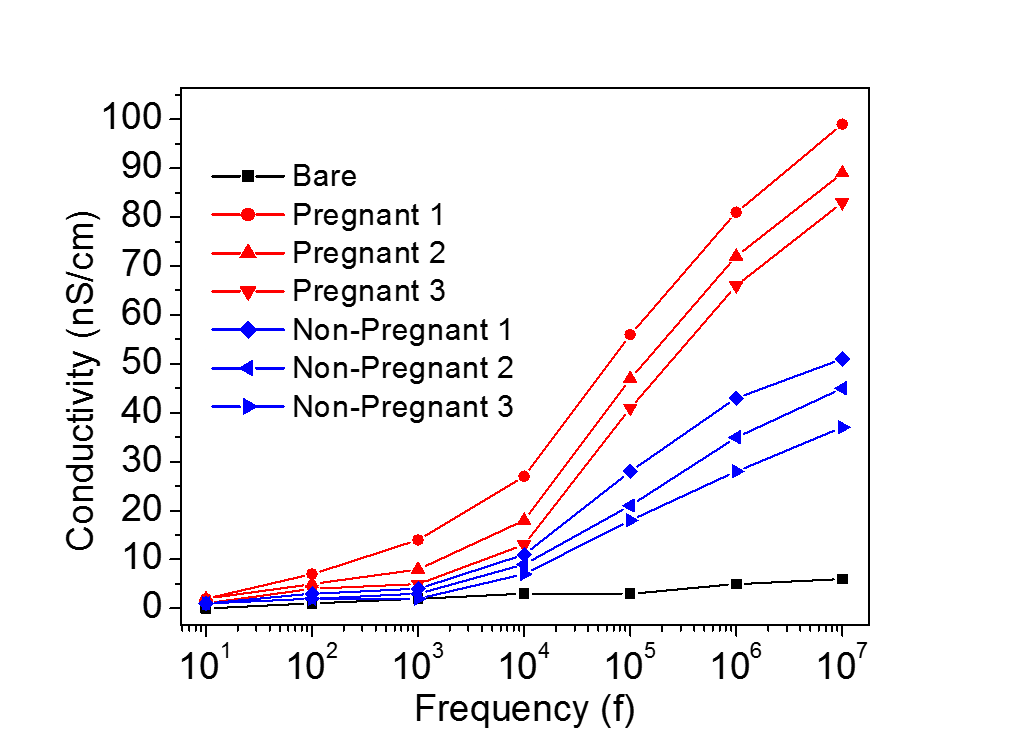


**Figure E.** Conductivity measurements for pregnant and non-pregnant women’s urine.
